# Editorial: Strength and conditioning in football: driving physical performance through research and innovation

**DOI:** 10.3389/fphys.2024.1437251

**Published:** 2024-06-06

**Authors:** Marco Beato, Chris J. Bishop, Anthony N. Turner

**Affiliations:** ^1^ School of Allied Health Sciences, University of Suffolk, Ipswich, United Kingdom; ^2^ Faculty of Science and Technology, London Sport Institute, Middlesex University, London, United Kingdom

**Keywords:** soccer, training, technologies, performance, testing

## Introduction

Contemporary sports rely on scientific research and technological advancements to enhance performance and promote health (Paul et al., 2016; Beato et al., 2021; Randell et al., 2021). This is particularly evident in football (Bangsbo et al., 2006; Turner and Stewart, 2014; Beato et al., 2024), as the world’s most popular sport. Frontiers in Physiology and Frontiers in Sport and Active Living recognize the critical role of strength and conditioning; thus, this Research Topic entitled “*Strength and conditioning in football: driving physical performance through research and innovation*” was designed to promote dialogue and disseminate knowledge within the sports science community. Strength and conditioning refers to the planning, delivering, monitoring, and reviewing of specialized physical training programs designed to enhance athletic performance, reduce the risk of injuries, and optimize fitness for football players (Cormie et al., 2011; Jeffries et al., 2022; Bishop et al., 2023; Turner, 2024). Consequently, our aim was to publish a series of studies that showcase the latest methodological advancements in strength and conditioning for football. We were particularly interested in innovative exercises that enhance adaptive responses and studies that explore effective ways to integrate new technologies, monitoring tools and testing protocols.

This Research Topic of Frontiers in Physiology and Frontiers in Sports and Active Living, contains 11 manuscripts meeting the editorial criteria, including 10 original research articles (Chmura et al.; Chmura et al.; Skala and Zemkova; Asencio et al.; Branquinho et al.; Paravlic et al.; Pimenta et al.; Rocha et al.; Szabo et al.; Zhai and Qin) and 1 brief research report Asimakidis et al.

Chmura et al. aimed to investigate the effects of high-intensity interval training and the anaerobic and psychomotor fatigue thresholds on physiological parameters in young soccer players. They found that both thresholds shifted toward higher loads and the proposed specific high-intensity interval training effectively increased the exercise capacity of soccer players. Practitioners could use these high-intensity interval training methods to effectively increase their players’ physical capacities.

Foqha et al. aimed to assess the effects of 10 weeks of FIFA 11+ training on the physical performance of elite seven-a-side soccer players. They found that the 10-week FIFA 11+ program resulted in notable enhancements in acceleration and agility performance compared to standard training for elite seven-a-side soccer players. These favourable results warrant additional research on implementing and optimizing the FIFA 11+ program, offering valuable guidance to coaches and athletes aiming to maximize its benefits in real-world scenarios.

Skala and Zemkova, aimed to investigate the neuromuscular and perceptual-cognitive response to small-sided games, and the relationship between pre- and post-small-sided games performance and exercise load in youth soccer players. They found that small-sided games induced fatigue that impacts planned and reactive agility, decision-making, and explosive strength in youth soccer players, irrespective of internal or external load variables.

Paravlic et al. aimed to investigate the associations between bilateral performance utilizing countermovement jump, squat jump, speed, unilateral jump, isokinetic peak torque in knee extension and flexion, and tensiomyography parameters. They also investigated whether the asymmetries derived from unilateral tests are associated with bilateral tests in elite female soccer players. The authors found several significant, albeit inconsistent, correlations between the diverse performance scores obtained highlight the necessity for a multifaceted and thorough diagnostic strategy in female soccer players.

Pimenta et al. compared the average speed, knee flexor peak torque, and shear modulus of the hamstrings after a repeated sprint task among football players at various competitive levels and playing positions. Surprisingly, the study found that neither the average sprint speed performance parameter nor the mechanical parameters were effective in distinguishing football players based on their competitive levels or positions on the field.

Szabo et al. aimed to examine the effect of a 10-week intervention with the TOCA Football System tool (which is a high repetition technical toolkit practice) and training method on elite youth athletes’ sport-specific motor skills and anthropometric variables. They found that this intervention using the TOCA Football System was safe and did not negatively impact athletes’ performance. Some significant improvements were observed within groups; however, no significant differences were found between the intervention and control groups.

Branquinho et al. aimed to investigate the ideal training load to be applied during periods of fixture congestion to ensure an adequate dose-response effect for performance maintenance. During a busy season for an elite Brazilian professional team, a positive training load was observed. However, the interference effect arises when high physical training is applied to various skills (such as change of direction and straight-line running) throughout the season. Additionally, the regression tree model proves valuable for identifying optimal loads and potential corrections to enhance athletes’ match performance.

Rocha et al. aimed to investigate whether the use of transcranial direct current stimulation on the primary motor cortex improves the performance of soccer players. However, the authors did not find any change in the vertical jump performance as well no improvement in subjective scales (e.g., pain, recovery and rating of perceived exertion). New studies should be developed with different stimulus intensities in different cortical areas and sports modalities, to better understand the effect of transcranial direct current stimulation on soccer performance and subjective scales.

In a study by Zhai and Qin, the impact of traditional resistance training (e.g., squat and deadlift) *versus* complex training on physical and technical performance in amateur futsal players was investigated. Over an 8-week intervention, players from two amateur futsal teams followed different training protocols. The findings suggest that complex training could enhance specific performance parameters, including strength and power, more effectively than resistance training for amateur futsal players.

In their study, Asencio et al. compared the effects of two flywheel resistance training programs: variable intensity and constant intensity. Seventeen amateur footballers were divided into these two groups, both with equal training volumes. While both groups showed similar improvements in one-repetition maximum strength, the constant intensity group exhibited greater enhancements in the 10-m sprint. However, no significant differences were observed in countermovement jump, change of direction, or 30-m sprint performance following the protocols. Further research is necessary to fully understand the distinctions between constant and variable intensity flywheel resistance training.

Asimakidis et al. provided insight into the current fitness testing practices in elite male soccer. One hundred and two practitioners from professional soccer leagues across 24 countries completed an online survey. The authors reported that the scientific literature influences test selection, but practical constraints and professional experience also play a role. Practitioners test less frequently than they consider optimal due to time and competitive schedules. Pre-season is the most common time for fitness testing, while competitive periods leave less time. A “hybrid” approach, combining standalone and integrated testing, may address this Research Topic. Microsoft Excel is the preferred software for data analysis. Finally, the survey suggested that tailored visualizations are more common for coaches and players—the key distinction is that coaches often receive more detailed information than players.

## Final considerations

The editors of this Research Topic emphasize the need for further research on the physiological mechanisms and adaptations resulting from strength training in football players. Specifically, they highlight the importance of studying youth and female populations, as well as examining ecological contexts with professional male and female players. Such research could significantly enhance the adoption of strength and conditioning training methods in practical settings. The 11 included articles provide evidence and insights into key aspects of strength and conditioning, potentially informing the implementation of new technologies and inspiring fresh research ideas.
